# Short Stature in Chronic Kidney Disease Treated with Growth Hormone and an Aromatase Inhibitor

**DOI:** 10.1155/2015/738571

**Published:** 2015-05-25

**Authors:** Susan R. Mendley, Fotios Spyropoulos, Debra R. Counts

**Affiliations:** ^1^Department of Pediatrics, University of Maryland School of Medicine, Baltimore, MD 21201, USA; ^2^Department of Medicine, University of Maryland School of Medicine, Baltimore, MD 21201, USA; ^3^Department of Pediatrics, University of Iowa Children's Hospital, Iowa City, IA 52242, USA; ^4^Department of Pediatrics, Sinai Hospital, Baltimore, MD 21215, USA

## Abstract

We describe an alternative strategy for management of severe growth failure in a 14-year-old child who presented with advanced chronic kidney disease close to puberty. The patient was initially treated with growth hormone for a year until kidney transplantation, followed immediately by a year-long course of an aromatase inhibitor, anastrozole, to prevent epiphyseal fusion and prolong the period of linear growth. Outcome was excellent, with successful transplant and anticipated complete correction of height deficit. This strategy may be appropriate for children with chronic kidney disease and short stature who are in puberty.

## 1. Introduction

Growth failure is a well-recognized complication of chronic kidney disease (CKD) and if left untreated can adversely affect quality of life [1]. In addition to correction of metabolic acidosis, secondary hyperparathyroidism, and vitamin D deficiency, recombinant human growth hormone (GH) is the most effective therapy to improve growth velocity [2]. However, these strategies may be insufficient in older children who come to attention late with advanced CKD and significant short stature. Advancing puberty and resulting epiphyseal closure may limit the time available for linear growth. We present an alternative approach using an aromatase inhibitor, anastrozole, which has been shown to be effective in idiopathic short stature but has not, to our knowledge, been utilized to maximize height potential in growth failure of CKD.

Inhibition of aromatase activity was initially recognized as beneficial in the therapy of estrogen-sensitive breast cancer. The aromatase enzyme complex, which is formed by cytochrome P450 XIX (CYP19) and the nicotinamide-adenine dinucleotide phosphate cytochrome P450 reductase, is differentially expressed in various tissues. Its role is to aromatize the steroid A ring of androgens (androstenedione and testosterone) resulting in the peripheral conversion of androgens to estrogen as well as the conversion of estrogen to catechol estrogen, 2-hydroxyestrogen, and 6*α*-hydroxyestrogen [3–5]. The importance of this process to regulating longitudinal growth was recognized in 1994-1995 in reports of two men with tall stature and unfused epiphyses who still manifested adult pubertal development. Further insight into the effect of estrogen on skeletal maturation was demonstrated when these patients were treated with estrogen replacement therapy which led to epiphyseal fusion only in patients with aromatase deficiency [6–8]. Therapeutic use of aromatase inhibition to increase predicted adult height has been shown to be effective in idiopathic short stature [9] although its use remains limited and awaits further study [10]. Despite limited data, the circumstances of this case suggested a benefit to the use of anastrozole to delay epiphyseal fusion in order to prolong linear growth.

## 2. Case Report

A 14-year-old male was evaluated for short stature and no previous medical problems were known. Height was 146 cm (SDS score −2.17) and weight was 34.8 kg (SDS score −2.30). There was no lower extremity bowing. He was Tanner 1 with bone age 12 6/12 years and predicted adult height was 169 cm (SDS score −1.20) and his midparental height was 174 cm. Both parents reported normal growth pattern with normal timing of puberty. Evaluation revealed serum creatinine of 3.6 mg/dL and serum bicarbonate of 18 mmol/L. Intact parathyroid hormone (PTH) was 188 ng/L. Upon further evaluation, ultrasonographic and radiologic studies demonstrated bilateral hydroureter and hyperechoic kidneys with right grade 5 reflux and left grade 4 reflux, leading to a presumptive diagnosis of longstanding reflux nephropathy.

**Figure 1:**
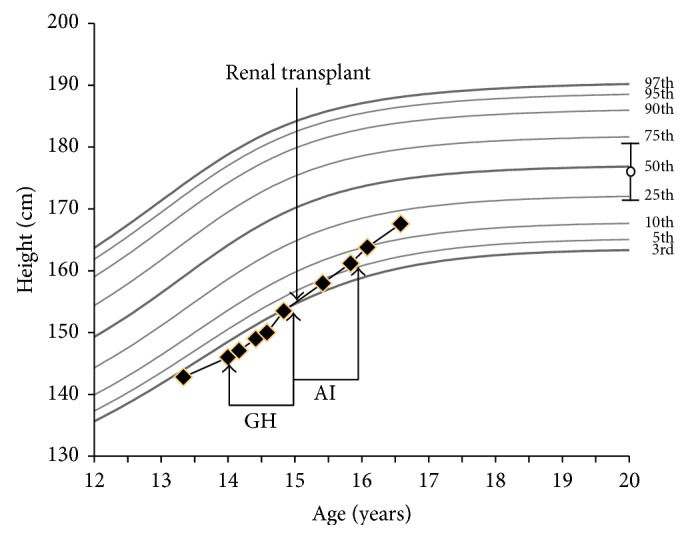
Observed growth curve of subject with prediction of final height. GH: interval of growth hormone therapy with growth velocity of 8.5 cm/year; AI: duration of aromatase inhibitor (anastrozole) therapy with growth velocity of 9.8 cm/year. Annualized growth velocity after AI therapy = 5.4 cm/year. Circle with vertical bars denotes predicted adult height by the method of Greulich and Pyle [10].

Initial therapy included sevelamer, sodium citrate, erythropoietin, calcitriol, and GH (0.053 mg/kg/day). There was good control of acidosis and metabolic bone disease with intact PTH maintained between 131 and 177 ng/L. Delayed puberty was treated with a short course of low dose testosterone (50 mg monthly IM for 4 months) in order to improve GH response, which was followed by spontaneous puberty. Annualized growth velocity was 8.5 cm/year (Figure 1), but renal function worsened and the patient was prepared for renal transplantation one year after presentation. At that time, he had Tanner 3 pubic hair and Tanner 2 genitals with a testosterone level of 12.1 nmol/dL and had achieved a height of 155.6 cm (SDS score −1.80). GH was discontinued at the time of transplant because we anticipated early use of glucocorticoids for immunosuppression, which would decrease GH therapy efficacy, and correction of renal function which could make GH therapy unnecessary. Since time for growth became limited by the need for transplant and initial steroid treatment, therapy with an aromatase inhibitor (anastrozole 1 mg daily) was initiated to delay epiphyseal fusion and provide additional time for linear growth. Despite clinician's request, a bone age was not obtained at this time. Transplant immunosuppression included basiliximab induction followed by tacrolimus, mycophenolate mofetil, and glucocorticoids with excellent allograft function. Serum creatinine normalized to 1.0 mg/dL. Prednisone was tapered to 0.1 mg/kg/day by month five and to 0.1 mg/kg every other day by month nine with stable kidney function and no signs of rejection. Ultrasensitive estradiol was <11 pmol/L demonstrating adequate blockade of testosterone conversion. Annualized growth velocity was 9.8 cm/year after transplant (Figure 1) so GH was not restarted. Anastrozole was continued for a total of 12 months and then was stopped when height was 163.8 cm (SDS −1.509), bone age was 14 years, and predicted adult height, by the method of Greulich-Pyle [11], was 177 cm (SDS = 0). Patient has continued to exhibit spontaneous pubertal progression after transplant, and annualized growth velocity off anastrozole and growth hormone therapy was 5.7 cm/yr.

## 3. Discussion

The age of onset of CKD and the degree of residual renal function are highly correlated with the degree of growth retardation, making early intervention crucial in order to optimize the height outcome and achieve a near normal final height [12]. Current guidelines [13] recommend that acidosis and metabolic bone disease be treated and nutritional deficiencies be corrected and if growth velocity or height SDS score remains abnormal, treatment with recombinant GH should be considered. This was our plan when the patient first presented, but we were concerned that impending puberty would limit our success.

Management of patients who are at pubertal age with significant short stature is challenging. Pubertal hormones lead to advancement of bone age and fusion of the epiphyses, limiting the time available for linear growth. However, there is also an important stimulant effect of testosterone on linear growth which enhances the efficacy of exogenous growth hormone [14, 15] and the combined therapy was initially chosen in our patient to maximize growth velocity as well as promote age-appropriate secondary sexual characteristics. We recognized the possibility that testosterone administration and subsequent pubertal progression might later limit growth duration but were reassured by the availability of anastrozole therapy to delay epiphyseal fusion. While both androgens and estrogens promote epiphyseal growth, estrogens are the major cause of epiphyseal fusion. Aromatase inhibitors have been utilized as adjuvant treatment for boys with disorders of growth of a variety of causes, aiming to extend the duration of linear growth by delaying epiphyseal fusion. Their use increases predicted adult height in boys with GH deficiency, idiopathic short stature, and constitutional delay of growth [9, 16, 17]. This is the first patient reported to our knowledge with growth failure secondary to CKD who has received anastrozole and we wonder whether there is something different about growth in this setting which would make the treatment particularly beneficial. Such a question would require additional study in a larger group of patients and we hope our report is an impetus to this.

An alternative strategy to delay epiphyseal fusion and increase final height might have been to suppress puberty with a gonadotropin (GnRH) analogue. In contrast to GnRH analogues, aromatase inhibitors do not delay the natural progression of secondary sexual characteristics in boys and are less likely to cause adverse psychosocial effects of suppressed puberty. Also, the effectiveness of combined GH and GnRH analogue in patients with short stature is equivocal and a recent consensus statement did not suggest their routine use for short stature [18]. By contrast, there is evidence that aromatase inhibitors increase predicted adult height in adolescent males with a good safety profile [19]. Consistent with its relatively low potency as an inhibitor of cytochrome P450 3A at therapeutic concentrations [20], anastrozole appeared to have no significant effect on tacrolimus levels or renal allograft function in our patient. Since aromatase inhibitors are administered orally rather than by injection, patient acceptance is generally good.

Our patient appears to have completely corrected his large height deficit (using predicted adult height as treatment outcome, shown in Figure 1) with several therapies that were used in combination and in sequence, including correction of metabolic abnormalities, testosterone, 12 months of growth hormone followed by 12 months of anastrozole therapy, and a rapid steroid taper after transplantation. Although the use of anastrozole is a promising measure in this setting we cannot be confident which of the above treatments had the greatest effect. His current treatment with low dose alternate day prednisone and a well-functioning renal allograft appears sufficient to achieve a growth trajectory similar to that which would have occurred in the absence of renal disease. Additional follow-up until near final height will be important to judge the efficacy of these interventions, since predicted height may overestimate true height outcome. In this patient we utilized sequential GH and aromatase inhibitor therapy, but we can foresee a circumstance where combined therapy might be appropriate, depending upon a patient's pubertal status and transplant or dialysis plan. In summary, treatment with GH and an aromatase inhibitor may provide unique benefits in the setting of advanced CKD and should be considered in children who present with significant short stature close to puberty when time for other therapeutic options is limited.
